# Long Noncoding RNA LINC00526 Represses Glioma Progression via Regulating miR-5581-3p/BEX1

**DOI:** 10.1155/2021/8171250

**Published:** 2021-02-04

**Authors:** Jian Yan, Youping Li, Chunhua Xu, Bin Tang, Shenhao Xie, Tao Hong, Erming Zeng

**Affiliations:** Department of Neurosurgery, The First Affiliated Hospital of Nanchang University, Nanchang 330006, China

## Abstract

The roles of long noncoding RNAs (lncRNAs) in regulating glioma progression have been widely recognized in recent years. This work was to investigate the roles and associated mechanisms of LINC00526 in glioma progression. LINC00526 expression in glioma tissues and cells and their normal counterparts was measured with quantitative real-time polymerase chain reaction method. Functions of LINC00526 in glioma were investigated with *in vitro* experiments. Moreover, competitive RNA (ceRNA) theory was employed to understand mechanisms of action of LINC00526 in glioma. LINC00526 was found to be decreased in glioma tissues and cell lines compared with their normal counterparts. Silencing the expression of LINC00526 promotes, while forcing its expression, inhibits glioma cell growth and invasion. Mechanism analyses showed LINC00526 functions as a sponge for microRNA-5581-3p (miR-5581-3p) to regulate brain-expressed X-linked 1 (BEX1) expression and, in the end, affects glioma progression. Collectively, our study indicated LINC00526 serves as a tumor-suppressive lncRNA and directly regulates miR-5581-3p/BEX1 axis in glioma.

## 1. Introduction

Glioma is the major type of brain cancer [1]. Notwithstanding improvements in glioma treatment methods, including surgery, chemotherapy, and targeted therapy methods in recent years, the prognosis of glioma remains undesirable [2]. The obstacle is that mechanisms associated with glioma progression are still largely to be explored.

Long noncoding RNAs (lncRNAs) are a type of ncRNAs at the length over 200 nucleotides [3]. lncRNA revealed that it could stimulate or suppress cancer progression via the following manners: chromatin modification, mRNA splicing, and posttranscriptional modulation [4–6]. Competitive RNA (ceRNA) proposed by Salmena and coworkers at 2011 is a type of posttranscriptional modulation mechanism, which indicated lncRNA sponged microRNA (miRNA) to regulate messenger RNA expression [7].

LINC00526, a 1322 nucleotide long lncRNA, is located at chromosome 18p11.31. A previous work showed LINC00526 accompanied with ARHGDIB, IDH2, ARL14, GSTM2, and LURAP1 could be used as predictors for overall survival of bladder cancer patients [8]. Another work showed low LINC00526 expression was an indicator for poorer overall survival of glioma patients [9]. Besides that, LINC00526 was reported to interact with EZH2 to repress glioma progression [9]. However, the detailed mechanisms of LINC00526 in glioma remain to be further investigated.

This work was aimed to investigate the biological functions of LINC00526 in glioma. Bioinformatic analysis and rescue experiments tool showed that LINC00526 regulates microRNA-5581-3p (miR-5581-3p)/brain-expressed X-linked 1 (BEX1) axis to suppress glioma progression.

## 2. Materials and Methods

### 2.1. Ethical Approval

Paired tumor tissues (35) and normal tissues (35) were collected from patients who underwent treatment at the first affiliated hospital of Nanchang university and treated with RNA later before frozen in liquid nitrogen. All participants have provided written informed consent. The study protocol was approved by the ethics committee of the first affiliated hospital of Nanchang university. These tissues were stored in liquid nitrogen for further usage.

### 2.2. Cells

Glioma cells (U87 and U251) and human astroglial cell line of NHAs were purchased from Shanghai Cell Bank. RPMI-1640 medium (Invitrogen, Carlsbad, CA, USA) containing 10% fetal bovine serum (FBS, Invitrogen) was used to culture these cells at a 37°C moist incubator with 5% CO_2_.

### 2.3. Transfection

Small interfering RNA for LINC00526 (si-LINC00526), negative control (si-NC), miR-5581-3p mimic, and negative control (mi-NC) were synthesized by GenePharma (Shanghai, China). pcDNA3.1-LINC00526 (pLINC00526) and pcDNA3.1-brain-expressed X-linked 1 (pBEX1) were bought from Hanbio (Shanghai, China). Cells were plated in a 6-well plate, grown to 80 % confluence, and transfected with these molecules (25 nM miRNAs, 50 nM siRNAs, and 4 *μ*g expression constructs) using Lipofectamine 2000 (Invitrogen) for 48 h.

### 2.4. Quantitative Real-Time Polymerase Chain Reaction (qRT-PCR)

TRIzol reagent (Invitrogen) was used to isolate RNA sample from tissues and cells according to manufacturer's instructions. RNA concentration was quantified using NanoDrop-1000. First-strand cDNA was synthesized using HiScript SuperMix (Vazyme, Nanjing, China). cDNA sample was diluted and used for qRT-PCR analysis at ABI 7500 system (Applied Biosystems, Foster City, CA, USA) with SYBR Green (Takara, Dalian, China) in line with the provided protocols. Procedure used was as follows: predenaturation at 95°C for 30 s, 40 cycles of denaturation at 95°C for 10 s, annealing at 60°C for 20 s, and extension at 70°C for 10 s. 2 − ∆∆CT method was used to calculate relative gene expression level with GAPDH and U6 snRNA as internal controls. Primers used were LINC00526: 5′-TTCAGGCTTCTGGGTCTC‐3′ (sense) and 5′-TTCACGGTTGGTATTTCGG‐3′ (antisense); BEX1: 5′-CCTCCCTTTGGATGCTGGTGAAT-3′ (sense) and 5′-CTCATCCTTGCCTGTGGTTCTCC-3′ (antisense); GAPDH: 5′-GGTCTCCTCTGACTTCAACA-3′ (sense) and 5′‐GTGAGGGTCTCTCTCTTCCT-3′ (antisense); miR-5581-3p: 5′-CGTCTTGCAGGCCGTCATG-3′ (sense) and 5′-GCTGTCAACGATACGCTACCTA-3′ (antisense); U6 snRNA: 5′-GCTTCGGCAGCACATATACTAAAAT-3′ (sense) and 5′-CGCTTCACGAATTTGCGTGTCAT-3′ (antisense).

### 2.5. Cell Viability Assay

2,000 cells were plated in 6-well plate and incubated at above-described conditions for 0, 24, 48, and 72 h. 10 *μ*l MTT reagent (Beyotime, Haimen, Jiangsu, China) was added to each well for 4 h and then incubated with dimethyl sulfoxide. Cell viability was measured by measuring optical density (OD) value at 490 nm using microplate reader.

### 2.6. Flow Cytometry Assay

1  ×  106 cells were detached using trypsin, washed with PBS, and stained with 10 *μ*l Annexin V-FITC and propidium iodide (PI) in binding buffer at darkness for 10 min. Cell apoptosis percentage was detected by flow cytometry equipped with FACS Diva software. Cells at early and late apoptosis stage were both considered as apoptosis cells.

### 2.7. Transwell Invasion Assay

Transwell chamber with or without Matrigel-coated was used to detect cell invasion or migration ability, respectively. 2 × 105 cells in 300 *μ*l serum-free RPMI-1640 were plated into upper chamber, whereas 500 *μ*l RPMI-1640 containing 20% FBS was added into lower chamber. After 48 h incubation, migrated or invaded cells were fixed with methanol and dyed with crystal violet. Images from 5 random fields were captured under microscope to calculate migrated or invaded cell numbers per filed using formula: total migrated or invaded cell number of five fields/5.

### 2.8. RNA Immunoprecipitation (RIP) Assay

EZMagna RNA immunoprecipitation (RIP) kit was employed for RIP assay according to manufacturer's protocol. Cells were lysed in RIP buffer and incubated with magnetic beads conjugated with anti-IgG or anti-AgO2. After being washed with PBS and treated with proteinase K and RNase-free DNase I, pellets were treated with TRIzol reagent to isolate RNA sample.

### 2.9. Dual-Luciferase Activity Assay

The bioinformatic analysis tools predicted that there are interactions among miR-5581-3p with LINC00526 or BEX1, and therefore dual-luciferase activity assay was performed to validate this prediction. pGL3 (Promega, Madison, WI, USA) was used to build luciferase vectors by inserting wild-type sequence of LINC00526 or BEX1 into this vector (wt-LINC00526 or wt-BEX1). Mutant luciferase vectors contain the sequences of LINC00526 or BEX1 with miR-5581-3p binding sites mutated (5′-…GCAUGGA…-3′ to 5′-…cgAUccA…-3′) were generated from wild-type luciferase vectors with PCR methods using the sire-direct mutagenesis kit (Takara) according to the manufacturer's instructions and then sequenced to validate the sequences were mutated as desired. Cells were transfected with luciferase vectors and miRNAs using Lipofectamine 2000. Cells were collected to detect relative luciferase activity using dual-luciferase activity assay system (Promega) after 48 h of transfection.

### 2.10. Tumor Xenograft Assay

The glioma cells with LINC00526 stably expression (pLINC00526) or control (2 × 105 cells) were injected into right flank of nude mice (*n* = 5 for each group). Tumor volume was calculated every 7 days for 4 times by measuring the width and length of the tissues. Tumor volume was calculated using formula: tumor volume = length × width 2 × 0.5. After mice were sacrificed, tissues were excised to measure tumor weight. Study protocol was approved by ethics committee of the first affiliated hospital of Nanchang University and performed in accordance with the guide of care and use of laboratory animals.

### 2.11. Statistical Analysis

Data obtained from three independent experiments were analyzed at GraphPad Prism 7 (GraphPad. Software, Inc., USA) and displayed as mean ± SD. Differences were measured with Student's *t*-test (for two groups) or ANOVA assay and Tukey's post hoc test (for three or above groups). Differences were regarded as significant when *P* less than 0.05.

## 3. Results

### 3.1. LINC00526 Expression Was Decreased in Glioma

**Figure 1:**
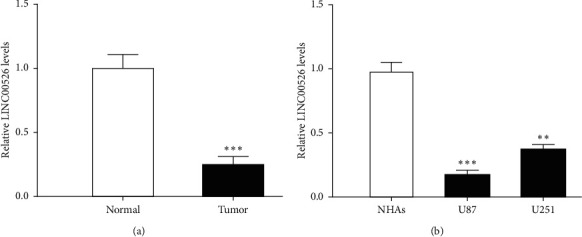
LINC00526 expression was decreased in glioma tissue cells. (a) Expressions of LINC00526 in glioma tissues and normal tissues. (b) Expressions of LINC00526 in glioma cells and the normal cells.

Firstly, we found the LINC00526 expression level was significantly decreased in glioma tissues compared with the matched normal tissues (Figure 1(a)). Furthermore, we found LINC00526 was expressed at a lower level in glioma cells than in normal cells (Figure 1(b)).

### 3.2. LINC00526 Inhibits Glioma Cell Proliferation and Invasion

**Figure 2:**
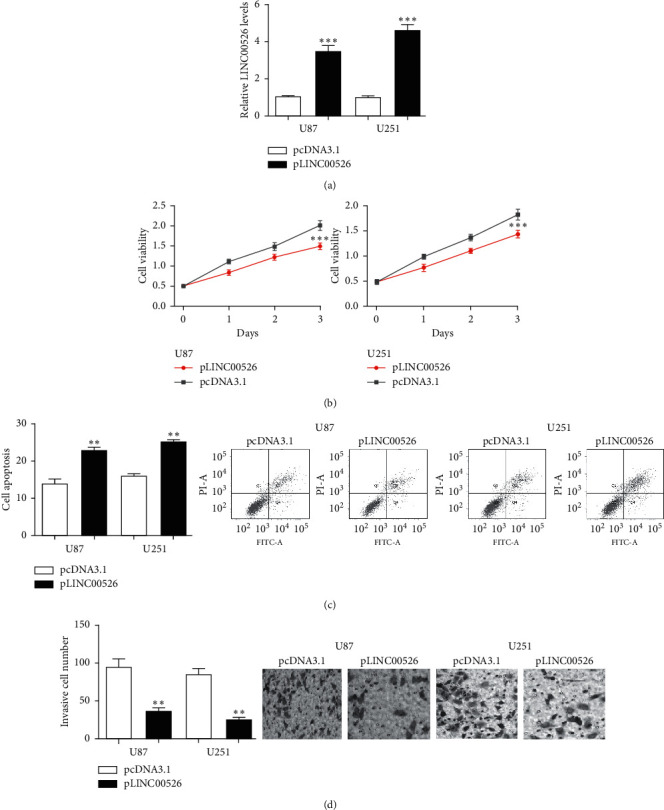
Overexpression of LINC00526 suppressed the viabilities and invasion of glioma cell lines. (a) LINC00526 level in glioma cells with transfection of pcDNA3.1 and pLINC00526. (b) MTT assay for glioma cells in transfection by pcDNA3.1 and pLINC00526. (c) Apoptosis rates for cells in transfection by pcDNA3.1 and pLINC00526. (d) Invasion abilities for cells in transfection by pcDNA3.1 and pLINC00526.

To explore the roles of LINC00526 in glioma, its expression was augmented by pLINC00526. As displayed in Figure 2(a), LINC00526 expression level was significantly increased by pLINC00526. Furthermore, MTT assay and colony formation assay showed LINC00526 overexpression inhibits glioma cell viability and promotes cell apoptosis abilities (Figures 2(b) and 2(c)). Moreover, we observed cell invasion ability was decreased by pLINC00526 (Figure 2(d)).

### 3.3. LINC00526 and BEX1 Shared the Binding Site in miR-5581-3p

**Figure 3:**
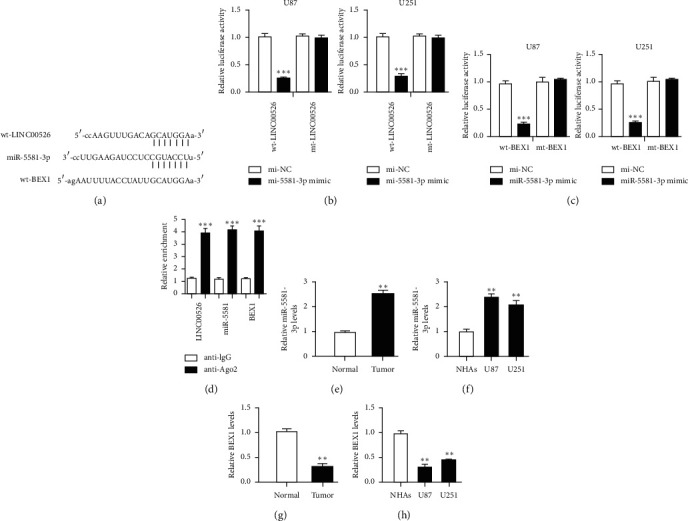
LINC00526 and BEX1 could bind with miR-5581-3p. (a) The shared binding sites for LINC00526 and BEX1 in miR-5581-3p. (b) Relative luciferase activity for glioma cells in transfection by mi-NC or miR-5581-3p mimic and wt-LINC00526 or mt-LINC00526. (c) Relative luciferase activity for glioma cells in transfection by mi-NC or miR-5581-3p mimic and wt-BEX1 or mt-BEX1. (d) RIP experiments for LINC00526, miR-5581-3p, and BEX1 coenrichment. (e) Expressions of miR-5581-3p in glioma tissues and normal tissues. (f) Expressions of miR-5581-3p in glioma cells and the normal cells. (g) Expressions of BEX1 in glioma tissues and normal tissues. (h) Expressions of BEX1 in glioma cells and the normal cells. miR-5581-3p: microRNA-5581-3p; wt: wild-type; mt: mutant; BEX1: brain-expressed X-linked 1; mi-NC: negative control miRNA.

Bioinformatic analyses showed miR-5581-3p contains binding site for LINC00526 and BEX1 (Figure 3(a)). To validate their potential interactions, luciferase activity reporter assays were performed. It was found miR-5581-3p mimic could decrease the luciferase activity in cells with wt-LINC00526 or wt-BEX1 transfection but not mt-LINC00526 or mt-BEX1 (Figures 3(b) and 3(c)). RIP assay indicated that LINC00526, miR-5581-3p, and BEX1 were coenriched in anti-Ago2 pellets (Figure 3(d)). To figure out the potential roles of miR-5581-3p and BEX1 in cancer progression, we explored their expression in cancer tissues and cells. We showed miR-5581-3p expression level was increased in glioma (Figures 3(e) and 3(f)). In addition, we demonstrated that BEX1 expression was decreased in glioma (Figures 3(g) and 3(h)).

### 3.4. LINC00526 Regulates BEX1 Expression through miR-5581-3p

**Figure 4:**
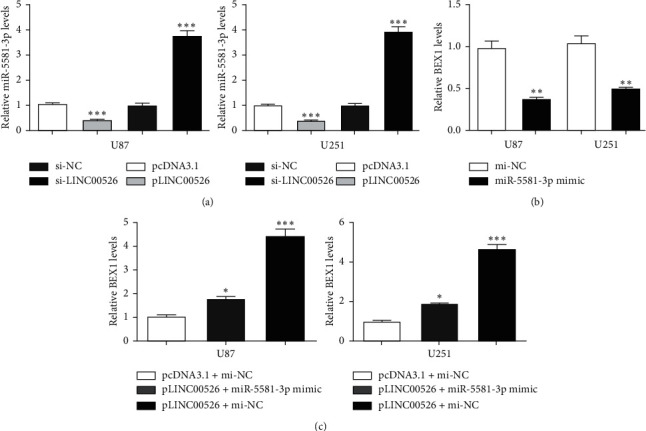
LINC00526 regulates BEX1 expression via miR-5581-3p. (a) Expressions of miR-5581-3p in glioma cells in transfection by pcDNA3.1, pLINC00526, si-NC, or si-LINC00526. (b) Expressions of BEX1 in glioma cells in transfection by mi-NC or miR-5581-3p mimic. (c) Expressions of BEX1 in glioma cells in transfection by pcDNA3.1 + mi-NC, pLINC00526 + miR-5581-3p, or pLINC00526 + mi-NC. microRNA-5581-3p; BEX1: brain-expressed X-linked 1; mi-NC: negative control miRNA; si-LINC00526: small interfering RNA targeting LINC00526; si-NC: negative control siRNA.

Next, we explored whether there is regulatory relationship between LINC00526 and BEX1. We showed miR-5581-3p expression level could be increased by si-LINC00526 and decreased by pLINC00526 in glioma cells (Figure 4(a)). Meanwhile, significant decrease of BEX1 expression was found after miR-5581-3p mimic transfection in glioma cells (Figure 4(b)). Additionally, we observed BEX1 expression was increased by pLINC00526, an effect could be partially reversed by miR-5581-3p mimic (Figure 4(c)).

### 3.5. BEX1 Inhibits the Proliferation and Invasion of Glioma Cells

**Figure 5:**
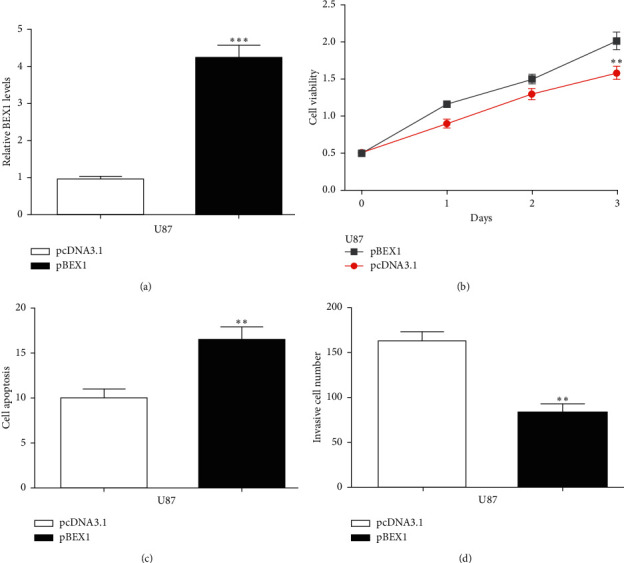
Overexpression of BEX1 suppressed the viabilities and invasion of glioma cell lines. (a) BEX1 level in glioma cells with transfection of pcDNA3.1 and pBEX1. (b) MTT assay for glioma cells in transfection by pcDNA3.1 and pBEX1. (c) Apoptosis rates for cells in transfection by pcDNA3.1 and pBEX1. (d) Invasion abilities for cells in transfection by pcDNA3.1 and pBEX1. BEX1: brain-expressed X-linked 1.

Next, we explored the effects of BEX1 in regulating glioma cell behaviors. We showed pBEX1 increased BEX1 expression level in glioma cells (Figure 5(a)). MTT assay, flow cytometry assay, and transwell invasion assay indicated that BEX1 overexpression could inhibit glioma cell proliferation and invasion (Figures 5(b)–5(d)).

### 3.6. LINC00526 Inhibits Tumor Growth *In Vivo*

**Figure 6:**
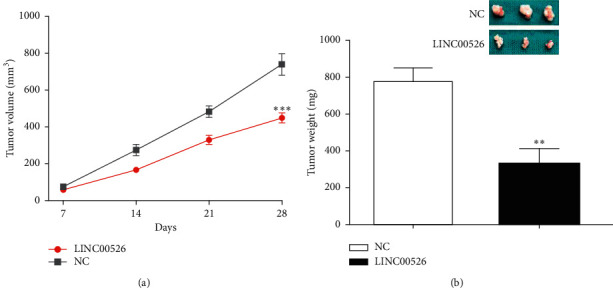
LINC00526 suppresses glioma growth *in vivo*. (a) Tumor volumes every seven days after LINC00526 overexpression. (b) Tumor weight was reduced after LINC00526 overexpression.

To explore the roles of LINC00526 *in vivo*, cells with stable LINC00526 overexpression were subcutaneously injected into nude mice. It was found LINC00526 overexpression could reduce tumor size and tumor weight (Figures 6(a) and 6(b)), which is in consistent with the *in vitro* analyses results.

## 4. Discussion

Increasing evidence indicates lncRNAs play crucial roles in cancers. lncRNA metastasis-associated lung adenocarcinoma transcript 1 could stimulate glioma metastasis through suppressing autophagy [10]. Another work showed LINC01140 could promote glioma progression via regulating miR-199a-3p/ZHX1 axis [11].

Here, we revealed LINC00526 expression was decreased in glioma. Hence, with the knowledge that LINC00526 expression was reduced in glioma, we then overexpress it in the glioma cells. Functional assays indicated LINC00526 overexpression could suppress glioma cell proliferation and invasion. *In vivo* experiments showed forced LINC00526 expression could suppress tumor growth. These results provided new evidence to validate the findings of a previous work [9].

lncRNAs could serve as ceRNA for miRNA to affect target gene expression [7]. We showed miR-5581-3p was a target of LINC00526 through bioinformatic analysis, luciferase activity assay, and RIP assay. miR-5581-3p was previously found to be negatively regulated by LINC00961 in hepatocellular carcinoma and thus affect cancer progression [12]. Subsequently, BEX1 was confirmed as a target of miR-5581-3p in glioma. BEX1 belongs to the BEX family and is found abnormally expressed in cancers [13–15]. For example, low BEX1 was identified in esophageal squamous cell cancer and significantly correlated with large tumor size and late tumor stage [13]. Besides that, BEX1 overexpression could suppress acute myeloid leukemia cell growth and tumor formation [14]. Here, LINC00526 overexpression significantly increased, whereas miR-5581-3p overexpression decreased BEX1 expression. Furthermore, BEX1 overexpression could inhibit glioma cell growth and invasion. We provided evidence that BEX1 expression can be regulated by LINC00526 via sponging miR-5581-3p, which provided new mechanism for low expression status of BEX1 in glioma.

In summary, we revealed LINC00526 expression was reduced in glioma. LINC00526 could inhibit glioma tumor growth by regulating miR-5581-3p/BEX1 axis. These results indicated LINC00526 functions as a tumor-suppressive lncRNA in glioma.

## Data Availability

Data are available from the corresponding author upon reasonable request.
